# 

**Published:** 1887-08

**Authors:** 


AUGUST, 1887.

### THE AMERICAN JOURNAL

OF

### DENTAL SCIENCE.

EDITED BY

F. J. S. GORGAS, M. D., D. D. S.

PUBLISHED MONTHLY. $2.50 PER ANNUM.

VOL. 21 THIRD SERIES. NO. 4.

BALTIMORE:
SNOWDEN & COWMAN.

LONDON:
TRUBNER & CO., 60 PATERNOSTER ROW.

PAYABLE IN ADVANCE.

